# Supplementary material for: Sputnik Light and Sputnik V Vaccination Is Effective at Protecting Medical Personnel from COVID-19 during the Period of Delta Variant Dominance
Source: Vaccines (Basel). 2022 Oct 26;10(11):1804. doi: 10.3390/vaccines10111804 (PMC9696561; doi:10.3390/vaccines10111804)
Supplement: Supplementary file 1 [file vaccines-10-01804-s001.zip › vaccines-1946009-supplementary.pdf]

Supplementary

Sputnik Light and Sputnik V vaccination is effective in protecting medical personnel from COVID-19 during the period of Delta variant dominance

Gennady T. Sukhikh [1], Tatiana V. Priputnevich [1], Darya A. Ogarkova[2], Andrei A. Pochtovyi[2,3], Daria D. Kustova[2], Vladimir I. Zlobin[2], Denis Y. Logunov[2], Vladimir A. Gushchin[2,3,*] and Alexander L. Gintsburg[2,4]

Table S1. Cases distribution of the medical center's employers, depending on the vaccination status during the entire period (11/26/2020 – 02/08/2022).

Vaccination status	Cases	Non-cases	VE, % (95% CI) (1-RR)	(1-HR)
Unvaccinated	199	0		
One dose	22	115	83.9% (76.5% – 89.0%)	87.2% (80.1% - 91.8%)
Two doses	263	648	71.1% (68.0% - 73.9%)	89.2% (86.9% - 91.0%)
Three or four doses	1	39	97.5% (82.7% - 99.6%)	96.5% (75.0% - 99.5%)
At least one dose	286	802	73.7% (70.9% – 76.2%)	89.1% (86.9% – 91.0%)

Table S2. Age depending on case severity and vaccination status (M±SD (95% CI)

Severity	Unvaccinated (*n*=512)	Vaccinated (*n*=285)`	*p* (Student's T-test)
Mild	344 (99.4%)<br>43.37±12.17<br>(42.08 – 44.66)	221 (100%)<br>41.32±12.49<br>(39.66 – 42.97)	0.053
Moderate	151 (100%)<br>44.87±11.70<br>(42.99 – 46.75)	59 (100%)<br>44.81±13.81<br>(41.22 – 48.41)	0.977
Severe	17 (100%)<br>49.47±14.41<br>(42.07 – 56.88)	5 (100%)<br>48.20±6.14<br>(40.58 – 55.82)	0.852
p (ANOVA)	0.076	0.098	
